# Investigation of Magnetoelectric Sensor Requirements for Deep Brain Stimulation Electrode Localization and Rotational Orientation Detection

**DOI:** 10.3390/s21072527

**Published:** 2021-04-04

**Authors:** Mevlüt Yalaz, Günther Deuschl, Markus Butz, Alfons Schnitzler, Ann-Kristin Helmers, Michael Höft

**Affiliations:** 1 Chair of Microwave Engineering, Christian-Albrechts-Universität zu Kiel, 24143 Kiel, Germany; mh@tf.uni-kiel.de; 2 Department of Neurology, Christian-Albrechts-Universität zu Kiel, 24105 Kiel, Germany; g.deuschl@neurologie.uni-kiel.de; 3 Institute of Clinical Neuroscience and Medical Psychology, Medical Faculty, Heinrich Heine Universität Düsseldorf, 40225 Düsseldorf, Germany; markus.butz@hhu.de (M.B.); alfons.schnitzler@hhu.de (A.S.); 4 Department of Neurosurgery, Christian-Albrechts-Universität zu Kiel, 24105 Kiel, Germany; Ann-Kristin.Helmers@uksh.de

**Keywords:** magnetoelectric sensor, SQUID, MEG, deep brain stimulation (DBS), directional DBS electrode, magnetic field measurement, electrode localization, rotational orientation detection

## Abstract

Correct position and orientation of a directional deep brain stimulation (DBS) electrode in the patient’s brain must be known to fully exploit its benefit in guiding stimulation programming. Magnetoelectric (ME) sensors can play a critical role here. The aim of this study was to determine the minimum required limit of detection (LOD) of a ME sensor that can be used for this application by measuring the magnetic field induced by DBS. For this experiment, a commercial DBS system was integrated into a head phantom and placed inside of a state-of-the-art Superconducting Quantum Interference Device (SQUID)-based magnetoencephalography system. Measurements were performed and analyzed with digital signal processing. Investigations have shown that the minimum required detection limit depends on various factors such as: measurement distance to electrode, bandwidth of magnetic sensor, stimulation amplitude, stimulation pulse width, and measurement duration. For a sensor that detects only a single DBS frequency (stimulation frequency or its harmonics), a LOD of at least 0.04 pT/Hz${}^{0.5}$ is required for 3 mA stimulation amplitude and 60 μs pulse width. This LOD value increases by an order of magnitude to 0.4 pT/Hz${}^{0.5}$ for a 1 kHz, and by approximately two orders to 3 pT/Hz${}^{0.5}$ for a 10 kHz sensor bandwidth. By averaging, the LOD can be reduced by at least another 2 orders of magnitude with a measurement duration of a few minutes.

## 1. Introduction

Deep brain stimulation (DBS) is an elective surgical procedure in which electrodes are implanted in specific areas of the brain. It has become an evidence based therapy for Parkinson’s disease with fluctuating mobility [1,2] and has become applicable to treat many other diseases [3]. The stimulating electrodes constantly deliver electrical impulses to target brain regions to control abnormal brain activity. The impulses are generated by a neurostimulator that is implanted under the skin (below the clavicle) and is connected by extension wires to the electrodes. In current clinical practice, each patient receives an individualized stimulation setting in which specific parameters are set based on clinician experience and readjusted based on clinically observed outcome parameters, i.e., symptom suppression such as tremor reduction. Thus, programming of the implanted pulse generator (IPG) or neurostimulator is a time-consuming, iterative, and trial-and-error based process [4,5,6,7]. DBS continues to be the subject of intensive fundamental and clinical research [8,9]. The number of diseases for which DBS is emerging as a potentially effective therapeutic measure is steadily growing. Continuous technical advancements in DBS systems, as well as improvements in medical imaging techniques, are also contributing to the immense development potential of this treatment modality. The overall goal is to provide the patient with an optimal therapy.

One of the revolutionary developments in recent years has been the development of directional electrode technology with segmented contacts (split into three segments along the circumference of the electrode) to steer the stimulation volume in a predefined direction. This has shown to improve the therapeutic effect and lower side effect thresholds when compared to electrodes with standard ring contacts [10,11,12]. To exploit the full potential of this technology and enable image-guided directional stimulation, detailed knowledge of the precise location and orientation of the electrode in the anatomical brain structures is required. To date, no generally established means are available for non-invasive electrode localization and rotational orientation detection. Currently, detection approaches are all based on neuroimaging data, e.g., the fusion of postoperative computed tomography (CT) with preoperative magnetic resonance imaging (MRI) to localize the electrode [13,14] or the detection of artifact patterns from X-rays, CT, and rotational fluoroscopy to determine electrode orientation [15,16,17]. These neuroimaging techniques are all associated with various limitations, and have been discussed in detail in previous works [18]. Recently, our group has developed and presented a new non-invasive and non-radiative method to determine the position and the rotational orientation of a DBS electrode by using magnetic field measurements [19]. To date, this method has been tested on head phantoms with an in-house constructed measurement system with a single Fluxgate sensor (20 pT/Hz${}^{0.5}$ between 0 and 1 kHz) [18,20] and with a state-of-the-art magnetoencephalography (MEG) scanner in a clinical setting based on Superconducting Quantum Interference Device (SQUID) sensors (3 fT/Hz${}^{0.5}$ between 0 and 1660 Hz) [19]. To do this, the neurostimulator was programmed with the following electrode configuration settings:

- Bipolar non-directional electrode configuration (ring stimulation) with the activation of contacts at different electrode heights for electrode localization and
- bipolar directional electrode configuration with the activation of the tip of the electrode against an individual segmented contact for electrode orientation detection.

Subsequently, the magnetic field induced by the stimulation was measured around the phantom or patient’s head at multiple measurement points and the position and rotation of the electrode was inferred using suitable forward models and localization algorithms. Our method localized the DBS electrode with an accuracy of about 2.2 mm and determined its orientation with an accuracy of 11${}^{\circ }$, regardless of the electrode location in the phantom. These accuracies were mainly limited by the imprecise position estimation of the phantom with respect to the measurement points (SQUID sensors). This indicates that the accuracies in position and orientation detection can be significantly improved if the measurement procedure is enhanced, i.e., if the position of the phantom and the measurement points can be accurately transferred into a uniform coordinate system. New technical developments are moving towards cap-shaped MEG sensor arrays [21,22] that move with the patient’s head (i.e., no relative movement between head and sensors) and can be adapted to the individual’s head shape. It would have the potential to overcome this limitation and could become a breakthrough in neurology, as they would achieve sufficient accuracy for this application. This could help in interpreting observed stimulation effects and in guiding time-consuming stimulation programming. These new systems make use of significant advances in the development of magnetic sensors that have the potential to overcome the limitation of current generation of MEG devices. Optically pumped magnetometers (OPMs) have been demonstrated to have sensitivities approaching the gold standard, those of commercial SQUIDs (10 fT/Hz${}^{0.5}$ between 0 and 100 Hz) [23] making them suitable for OPM-MEG development. However, these sensors still have too narrow of a bandwidth for use in DBS applications due to the presence of much higher frequencies (stimulation frequency and harmonics). It is reported that ME sensor approaches offer a high potential for this application since the resonance frequency of an ME sensor can be tuned to the stimulation frequency or its higher harmonics to measure the artificial magnetic signal of DBS [24,25,26]. However, such resonant, narrow-band ME sensors measure only a single frequency component of the entire broadband DBS signal, therefore, higher sensor sensitivity is required. Surface acoustic wave (SAW) sensors, which operate over a wide bandwidth of 50 kHz, may be more promising because they can pick up all frequency components of the signal [27]. Therefore, a high sensor sensitivity, as provided by a SQUID sensor, is not required. The objective of this work is to provide the minimum requirements that the magnetic field sensors must meet in order to be used for electrode localization and electrode orientation detection. The main focus is on the required operating frequency bandwidth and detection limit of the sensor.

## 2. Materials and Methods

### 2.1. Experimental Design

**Figure 1:**
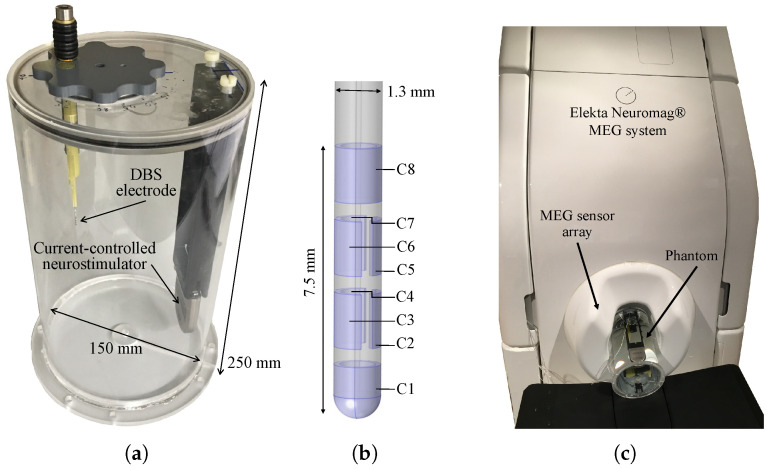
(**a**) The cylindrical phantom with the integrated deep brain stimulation (DBS) system which consists of a current-controlled neurostimulator and directional electrode by Boston Scientific. (**b**) The geometry of directional electrode and its contact numbers. (**c**) The phantom inside the MEG scanner.

The head phantom used in this study is depicted in Figure 1a. The cylinder body with dimensions that are comparable with a human head (diameter of 150 mm, height of 250 mm) was made of acrylic glass and was, therefore, neither electrically conductive nor magnetic. It was filled with an isotonic fluid (NaCl 0.9%) to mimic the electric conductivity of a human brain. We used a DBS system by Boston Scientific Inc. (Boston Scientific, Marlborough, MA, USA) consisting of a current-controlled DBS neurostimulator (Vercise™ PC) and a directional electrode (Versice Cartesia™) as used in clinical routines. This system is based on stimulation with traditional rectangular pulses including a stimulus pulse phase and a passive charge-balancing phase. It was integrated and fixed into the phantom. Moreover, we used non-magnetic material for all other components used in the phantom. The screws on top of the electrode holder were made of titanium, while the adjustment wheel and the holders for the electrode and neurostimulator casing were made of plastic. The structure of the directional DBS electrode is shown in Figure 1b. It is comprised of eight individually controlled platinum-iridium contacts (C1–C8), in which the two middle contact levels were segmented into three contacts each spanning 120${}^{\circ }$ of the circumference. Any combination of these contacts can be activated to steer the stimulation current direction. The outer jacket is made of polyurethane. MEG data were collected with an Elekta Neuromag VectorView^®^ MEG scanner at the Universitätsklinikum Düsseldorf (UKD, Düsseldorf, Germany) in Düsseldorf. Figure 1c depicts how the phantom was positioned within the MEG sensor array.

### 2.2. Data Acquisition

The MEG scanner was comprised of 306 individual sensors, corresponding to 102 magnetometers and 204 gradiometers arranged as 102 sensor triplets. Only the magnetometer data were used in this study. The noise level according to the datasheet was about 3 fT$/\sqrt{\mathrm{Hz}}$ for both magnetometers and gradiometers. The acquisition parameters of the MEG scanner were set as follows: The sampling rate was set to its maximum of 5 kHz. The low-pass filter was set to the highest possible value of 1660 Hz and the cut-off frequency of the high-pass filter was selected to direct current (no high-pass filtering) in order to obtain measurements of the DBS signal with the maximum allowed acquisition bandwidth. The duration of each measurement was three minutes. A total of nine measurements were taken (see Table 1) with different activated electrode contacts (1–8: contact numbers, first number: anode, second number: cathode) and stimulation parameters (amplitude, pulse width, frequency). The first three measurements were performed in bipolar mode with activation of contacts at different electrode heights with 3 mA of stimulation amplitude. The next three measurements were performed with 1.5 mA stimulation. The last three measurements were made under monopolar electrode configuration. The applied values (3 or 1.5 mA, 130 Hz, 60 μs) are values used in clinical routine. Since these were phantom measurements, the measured MEG signals were not contaminated by biological artifacts such as cardiac muscle, skeletal muscles, or eye movements. Thus, the measurements with a phantom represent an ideal case. The ambient noise solely consisted of the power line interference, which only affected non-important frequencies (50 Hz and harmonics). The distance between the electrode and MEG sensors varies between 7 cm (sensor closest to the electrode) and 15 cm (sensor farthest from the electrode). These distances are also to be expected in real patient measurements. The good quality of recorded data was ensured by an ‘empty room’ measurement prior to the start of the experiment and by visual inspection of approximately 1 min of MEG recording before each measurement.

### 2.3. Signal Processing

**Figure 2:**
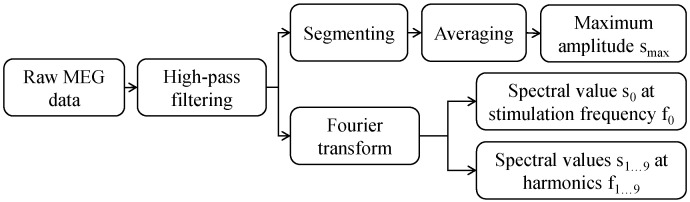
Each measured MEG signal is high-pass filtered with 60 Hz cutoff frequency, segmented, and averaged to a single DBS time period. Then, the maximum amplitude value is taken. The filtered time signal is also transformed into the frequency domain and the spectral values at stimulation frequency $f_{0}$ and at its harmonics $f_{1...18}$ are taken.

The processing steps of the MEG measurements are depicted in Figure 2. For each measurement, we obtained 102 time signals, one from each MEG sensor. The measured data was imported into MATLAB^®^ (Version R2018a) and preprocessed using the FieldTrip toolbox [28]. Each time signal was high-pass filtered (6th order Butterworth) with a cutoff frequency of 60 Hz and without signal loss, since the signal contains higher frequencies (stimulation frequency of 130 Hz and its harmonics). Each signal was divided into short segments of length equal to the inverse of stimulation frequency ($T_{s}=1/f_{s}=7.69$ ms). These segments were then averaged which improved the signal-to-noise ratio (SNR) by $\sqrt{N}$, where N=180s×130Hz=23,400. The maximum value from this averaged time segment is then taken for each MEG sensor, resulting in a total of 102 values representing the measured field distribution around the phantom. In addition, each high-pass filtered time signal was also transformed into the frequency domain using the Welch’s method [29] with a Hanning window, and the amplitude value was taken from the spectrum at 130 Hz stimulation frequency and at its harmonics. Since the MEG system measured at a sampling rate of 5 kHz, a total of 18 harmonics ranging from 260 to 2470 Hz could be considered, although only the first 11 harmonics ranging from 260 to 1560 Hz could be measured without attenuation due to the 1660 Hz low-pass filter used in the MEG device. Therefore, in addition to 102 values obtained from the time signal, each measurement was also represented by 102 values obtained from each of the 19 frequencies in the frequency signal. The maximum amplitude from the averaged time signal was composed of the total acquisition bandwidth, which was limited to 1660 Hz by the MEG system. This amplitude value can be interpreted as the result of a measurement with a magnetic sensor with 1660 Hz bandwidth. A single amplitude in the spectrum considers only a single frequency component of the DBS signal, so this amplitude value can be interpreted as the result of a measurement performed with a narrow-band sensor that is sensitive only at the corresponding frequency.

Since magnetic sensors always exhibit noise as a function of frequency, the term density spectrum was used in this paper when investigating the minimum required detection limit. The power density spectrum has as its physical dimension the squared physical unit of the observed quantity per Hz. The amplitude density spectrum, which is the square root of the power density spectrum, has as its physical dimension the physical unit of the observed quantity per $\sqrt{\mathrm{Hz}}$ [30]. In this work, the term measured quantity refers to a magnetic flux density to be measured with the physical dimension Tesla. For the case that an amplitude density spectrum has as physical unit the dimension of the measured quantity per $\sqrt{\mathrm{Hz}}$, it is called the noise level or the limit of detection (LOD) of the sensor with the unit T/$\sqrt{\mathrm{Hz}}$. This results from the quotient of the amplitude noise density [V/$\sqrt{\mathrm{Hz}}$] and the sensitivity [V/T] of the sensor. The frequency bandwidth of a sensor describes a white noise behavior between the lower and upper cutoff frequency with the same noise level or LOD value.

## 3. Results

### 3.1. Presence of Magnetic Flux Densities in the Time Domain

**Figure 3:**
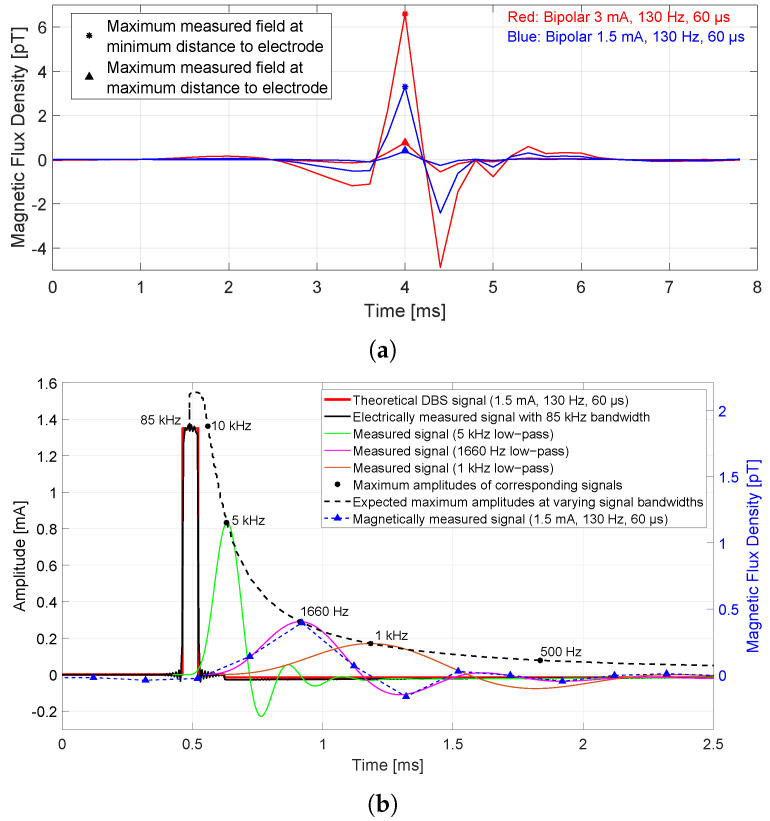
(**a**) The measured, preprocessed, and averaged time signal of a single DBS period for both bipolar configuration types, each considering an MEG sensor farthest from the electrode (small field) and a sensor closest to the electrode (larger field). The maximum amplitudes are marked with asterisk and triangle symbols, which represent the values with the highest SNR. (**b**) The DBS time signals in theory, electrically measured, and with different frequency bandwidths. The fewer frequency components there are in the signal, the smaller the maximum amplitude gets. The behavior of a magnetically measured signal exactly matches that of the electrically measured field with 1660 Hz low-pass.

The range of measured magnetic flux densities in the time domain is shown in Figure 3a for both applied bipolar electrode configurations. This figure demonstrates preprocessed and averaged signals of a single DBS period with 7.69 ms length (equals to the inverse of 130 Hz stimulation frequency), in which the maximum amplitudes were marked. For 3 mA bipolar mode, the maximum measured field at the sensor closest to the electrode (7 cm) was 6.6 pT and the maximum measured field at the sensor farthest away (15 cm) was 0.8 pT. All other measured field strengths were between these two values. For 1.5 mA bipolar mode, exactly half the values obtained at 3 mA were measured. The sampling rate of the MEG system limited the resolution of the signal over time. Marked amplitudes with asterisks and triangles in Figure 3a were entered in Table 2 under frequency bandwidth ‘0–1660 Hz’, because the acquisition bandwidth was limited by the MEG system at 1660 Hz. Measured results with monopolar electrode configuration were also added to the table but not included in the figure, since monopolar mode caused much larger fields (between 5 and 195 pT) that would stretch the figure. Figure 3b visualizes the result of how the maximum amplitude in time domain changed when sensors with different signal bandwidths were used. The set electrode configuration with 1.5 mA, 130 Hz, and 60 μs was measured electrically at the activated electrode contacts with 170 kHz sampling rate (black curve). A good agreement with the theoretical signal (red curve) can be seen, which had significantly more frequency components in the signal and thus perfectly represents the rectangular wave pulse. The maximum amplitude corresponds to 1.35 mA, since 90% of the applied stimulation amplitude is used for the stimulus pulse and 10% of the stimulation amplitude for the passive charge-balancing phase. The electrically measured signal was low-pass filtered with decreasing cut-off frequency and the maximum amplitude of the signal was taken in each case. Three additional curves are added to this figure which represent 5 kHz, 1660 Hz, and 1 kHz cut-off frequency. Maximum amplitudes were marked with black asterisks and, for more clarity, three more markers for 8 kHz, 10 kHz, and 500 Hz are added. It can be seen that the amplitude of the signal actually increases at very wide frequency bandwidths (10 to 85 kHz), but then as the bandwidth decreases, much fewer frequency components of the signal are considered which leads to amplitude reduction. For comparison, a magnetically measured signal (the exemplary signal measured at a large distance from the signals shown in Figure 3a was chosen) was inserted into a second y-axis. The pattern of the magnetically measured curve corresponded to the pattern of the electrically measured signal low-pass filtered with 1660 Hz. The maximum amplitude with 0.3 pT height changed when considering different sensor bandwidths; the amplitude became approximately 5 times larger with a 10 kHz bandwidth, and 1.6 and 17 times smaller with a 1 kHz and a 150 Hz bandwidth, respectively. The calculated values are given in Figure 2.

### 3.2. Presence of Magnetic Flux Densities in the Frequency Domain

**Figure 4:**
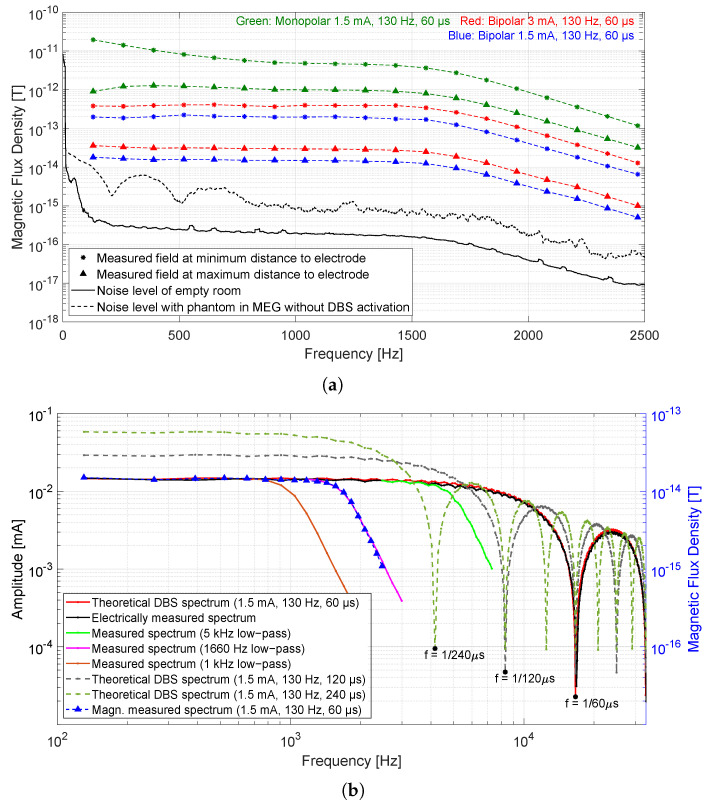
(**a**) The spectrum of the measured fields for the monopolar and both bipolar electrode configurations. The spectral values with 130 Hz stimulation frequency and at its corresponding harmonics are marked. In each case, an MEG sensor farthest from the electrode (small field) and a sensor closest to the electrode (larger field) were considered. (**b**) The DBS spectra in theory, electrically measured, and with different frequency bandwidths. The lower the bandwidth, the less frequency components of the DBS signal are considered. The behavior of a magnetically measured spectrum corresponds exactly to that of the electrically measured spectrum with 1660 Hz low-pass.

The range of measured magnetic flux densities in the frequency domain is illustrated in Figure 4a for the applied monopolar and for both applied bipolar electrode configurations. Markers represent the height of spectral values with a stimulation frequency of 130 Hz and the 18 corresponding harmonics ranging from 260 to 2470 Hz. The maximum frequency was limited by the sampling rate. The measured spectrum at the sensor closest to the electrode (7 cm) was marked by asterisks symbols and the spectrum at the sensor farthest away (15 cm) is marked by triangle symbols. All other measured spectra were between both markers. For 1.5 mA bipolar mode, exactly half the spectral values obtained at 3 mA were measured, i.e., 18 fT at the fundamental frequency for 1.5 mA stimulation and 36 fT for 3 mA stimulation. The decrease of spectral values after 1660 Hz is due to the system bandwidth. The flux densities in monopolar mode were much higher compared to the densities in bipolar mode with the same setting, this is because in bipolar mode, the reverse current in the connector and its corresponding wire canceled the field generated by the current flowing in the other direction, whereas in monopolar configuration, all current elements from the neurostimulator to the electrode within the cable and back within the saline solution contributed to the magnetic field. Therefore, the monopolar configuration could not be used to determine the position and rotation of the electrode. The measured spectral values at 130 Hz fundamental frequency corresponded exactly to the maximum amplitude in the 150 Hz low-pass filtered signal in the time domain. Corresponding values have been inserted in Table 2 under ‘130 Hz’. The spectrum of the ambient noise signal (with an equivalent noise bandwidth of 0.09) can be seen in Figure 4a. The corresponding measurement was performed without the phantom in the MEG. An increase in the noise floor was observed after placing the phantom into the MEG scanner without activating the stimulation current. This occurred because the neurostimulator was powered and was in stand-by-mode. Nevertheless, the background noise over all frequencies, especially over the frequencies of interest, was so low that the magnetic field generated by the stimulation was not affected. Figure 4b depicts the behavior of spectral values over the frequency of electrically measured, magnetically measured, and theoretical DBS signal. A good agreement between the theoretical signal (red curve) and the electrically measured signal (black curve) can be seen for frequencies up to 20 kHz. When the pulse width of stimulation increased to e.g., 120 or 240 μs, the magnitude spectrum loops became thinner and higher. In other words, the zeros move close to the origin (compare zeros at the frequencies 1/60, 1/120, and 1/240 Hz) and the spectral values became larger. Furthermore, the spectra of electrically measured signals with cut-off frequencies of 5 kHz, 1660 Hz, and 1 kHz were exhibited to this figure. The smaller the bandwidth, the fewer frequency components of the signal were measured. As a comparison, a magnetically measured spectrum (exemplary the spectrum measured at 15 cm distance to the DBS electrode as shown in Figure 4a is chosen) was inserted into a second y-axis. The course of the magnetically measured curve over the frequency corresponds to the course of the electrically measured spectrum low-pass filtered with 1660 Hz. At the fundamental frequency, a value of 18 fT was measured for 1.5 mA stimulation and 60 μs pulse width, which becomes twice and four times as large for double and quadruple pulse width, respectively.

### 3.3. The Required Frequency Bandwidth of Magnetic Sensor

**Figure 5:**
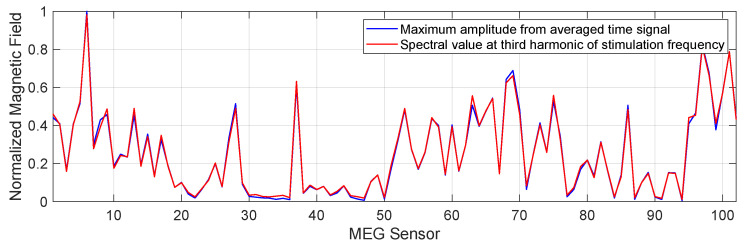
The comparison between normalized measured magnetic field values calculated in the time domain (maximum amplitude from averaged signal) and the frequency domain (spectral value at the third harmonic frequency of 130 Hz stimulation) for measurement number 1. The NRMS error between data is 0.07% and therefore negligible.

To determine the position and the rotational orientation of a DBS electrode in the phantom or patient head, the measurement data needed to be normalized to the maximum measured field closest to the electrode. In this manner, the magnetic field distribution around the phantom or patient head was normalized. This section investigates whether the behavior of the required magnetic field distribution across sensors depended on the frequency bandwidth of the sensor, i.e., whether the normalized behavior changed when more or less frequency components of the stimulation signal were measured. For that, we normalized the computed data of measurement number 1 in time and frequency domain according to Figure 2 across sensors. This allowed for a fair comparison and contrast of the data. Figure 5 provides the determined values for all 102 sensors obtained in time (by maximum value from averaged signal) and frequency domain (spectral value at third harmonic 520 Hz). The time values included frequency components up to 1660 Hz (limited by the MEG system), while the frequency values included only a single frequency component of the signal. The normalized root mean square error (NRMSE) between both data, which describes the quality of the similarity of both data, was 0.07% in this case. Errors in % for other frequencies 130, 260, 390, 650, 780, 910, 1040, 1170, 1300 Hz are 0.16, 0.18, 0.09, 0.07, 0.08, 0.09, 0.1, 0.11, 0.13 respectively and the average error is 0.11%. Thus, the difference in values obtained with respect to the frequency and time values was negligible, since a measurement accuracy of 0.5% showed no electrode localization displacement [18] and all of the calculated error values are below 0.5%. Nevertheless, slight differences of the errors in the figure could be described by the following points: resolution of digitized time signals during measurement, filtering of signals during digital signal processing, and amplitude spectrum estimation during frequency domain transformation. This result demonstrated that the same behavior of magnetic field distribution around the phantom could be observed either by considering maximum amplitude in the time domain or single spectral amplitude at one of the DBS frequencies. Thus, for the determination of electrode position and rotation, the magnetic field generated by DBS could be measured both with a narrow-band sensor sensitive only at one of the DBS frequencies and with a broadband sensor with a higher bandwidth. However, it should be noted that the signal amplitudes became drastically smaller with decreasing bandwidth (see previous section) and thus the noise level of the sensor must have been significantly improved. This is part of the next section.

### 3.4. The Required Limit of Detection

**Figure 6:**
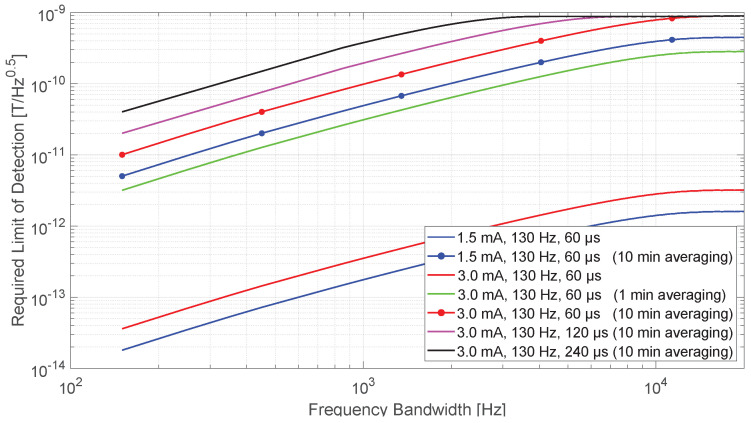
The required limit of detection (LOD) curves with respect to frequency bandwidth of a sensor for different bipolar electrode configuration types and measurement duration. As an example, the minimum required LOD with 150 Hz bandwidth is around 20 fT/Hz${}^{0.5}$ for 1.5 mA amplitude, 60 μs pulse width, and 130 Hz frequency without averaging. The noise level of a sensor can be 1 nT/Hz${}^{0.5}$ between 130 Hz and 10 kHz for 3 mA amplitude, 60 μs pulse width, and 130 Hz frequency with 10 min of averaging.

This section investigates what minimum limit of detection (LOD) a sensor must have to measure the magnetic field produced during stimulation in order to perform electrode localization and rotational orientation detection. White noise was assumed for sensors so that the LOD value was identical for all spectral components. Since flicker noise (1/f-noise) is only noticeable at low frequencies and the first frequency of interest is the much higher stimulation frequency, this assumption represented a good approximation. For an even more detailed investigation, however, one should consider the LOD frequency behavior of a real sensor. The blue curve in Figure 6 shows the increase in measured maximum signal amplitude in double logarithmic scale for the bipolar electrode configuration with 1.5 mA amplitude, 130 Hz stimulation frequency, and 60 μs pulse width, and represents the same curve in black dashed line from Figure 3b. This curve was chosen as the reference curve for LOD investigations because it represented the expected amplitudes at maximum distance from the DBS electrode; and for accurate electrode localization, all magnetic fields around the phantom or patients’ head should be measured, including more distant attenuated fields such as those from the curve. It resulted in a signal-to-noise ratio (SNR) of one. According to this curve, a LOD of at least 0.02 pT/Hz${}^{0.5}$ was required if the sensor had an operating bandwidth that detected only the fundamental frequency component of the stimulation signal, e.g., if the sensor was sensitive between 100 and 150 Hz. The LOD requirement decreased by an order of magnitude to 0.2 pT/Hz${}^{0.5}$ for a sensor with 1 kHz bandwidth and by about two orders of magnitude to 1.5 pT/Hz${}^{0.5}$ for 10 kHz bandwidth. The LOD curve could be correspondingly degraded by a factor of two (red curve) for twice the stimulation amplitude if it could be administered to the patient, since increasing the stimulation amplitude led to a proportional increase in the magnetic field. The LOD curve could be further increased by longer recording and averaging. A measurement of 1 minute resulted in a total of 130 Hz × 60 s = 7800 DBS periods and 10 minutes in 130 Hz × 60 s × 10 = 78,000 periods, which can be averaged. Since noise occurred stochastically, the standard deviation of the noise signal only grew by a factor of $\sqrt{N}$ when *N* single time periods were summed, and in relation, the signal grew by a factor of *N*. The SNR related to the signal amplitudes increased by $N/\sqrt{N}=\sqrt{N}$, which follows from the central limit theorem. Therefore, for 3 mA stimulation amplitude, a narrow-band sensor (sensitive at the fundamental frequency) could have a LOD of approximately 3 pT/Hz${}^{0.5}$ for 1 min of recording (dark green) and approximately 10 pT/Hz${}^{0.5}$ for 10 min of recording (red dashed). According to our physicians, a measurement time of 10 min in the MEG scanner is reasonable for the patient. The magnetic field could be increased again by a factor of 2 or 4 in the low frequencies when the stimulation pulse width was increased from 60 μs to 120 or 240 μs, however, only few patients would tolerate this. The corresponding LOD could thus be reduced for sensors with narrow and limited bandwidth (magenta curve and black curve), whereas the pulse width for the LOD had no effect for sensors with a bandwidth of 10 kHz or more.

## 4. Discussion

In this paper, the required sensing characteristics of magnetic field sensors needed to measure the magnetic field induced by DBS were investigated in order to determine the position and rotation of a directional DBS electrode. The work focused mainly on the minimum requirements to answer the question of what minimum frequency bandwidth and minimum detection limit a sensor must have so that the magnetic field remains measurable at measurement points at a realistic distance from the electrode. The investigations were performed in a state-of-the-art SQUID-based MEG scanner. SQUID sensors are still the gold standard of measuring magnetic fields, i.e., the most sensitive magnetic field sensors, with which resolutions in the femto Tesla range can be achieved. Such a high sensitivity is not necessary for this application because the stimulation current generates a magnetic field much larger than the magnetic field generated by human brain activity. Thus, this application represents a potential area for future sensor development, (magnetoelectric or surface acoustic wave sensors) by both industry and research institutes.

Our recently presented method [19] for determining electrode position and electrode rotation with magnetic field measurements is intended to provide an alternative to conventional neuroimaging techniques. The neuroimaging approach localizes the DBS electrode by visual inspection via metal artifacts of the electrode contacts on postoperative CT or MRI. The rotation of the electrode is determined by visual inspection over artifacts created by the radiopaque marker over the electrode contacts on X-rays, standard CT, flat-panel CT, or rotational fluoroscopy. All currently available methods expose the patient to radiation. The method based on magnetic field measurements is harmless (non-radiative and non-invasive) and safe for the patient. This simply involves programming and setting up the neurostimulator with the bipolar electrode configuration and appropriate choice of stimulation parameters to generate a defined magnetic field. The magnetic field distribution can then be measured around the patient’s head or phantom and used with precise forward modeling and localization algorithms to derive electrode position and rotation.

The investigations in this paper were performed with SQUID measurements in a clinical MEG scanner using a head phantom with dimensions comparable to a human head and clinically used DBS stimulation parameters. First, the presence of the magnetic field was analyzed in both the time domain and the frequency domain. The following observations were made:

- For 1.5 mA, 130 Hz, and 60 μs bipolar mode, the maximum measured field in the time domain at the sensor closest to the electrode (7 cm) was 3.3 pT and the maximum measured field at the farthest sensor (15 cm) was 0.4 pT. The measured amplitude in the frequency domain at the stimulation frequency was 0.2 pT for the sensor closest to the electrode and the spectral value at the sensor farthest away was 18 fT.
- An increase in the stimulation amplitude from 1.5 to 3 mA (with pulse width and frequency remaining the same) leads to a proportional increase in the magnetic field amplitude in both the time and frequency domain.
- The maximum amplitude of the magnetic field in the time domain (that is generated by the broadband stimulus signal) depends on the given frequency bandwidth of the sensor or system. Since the SQUID sensor acts like a low-pass filter due to the system frequency bandwidth (0–1660 Hz limited by the MEG system), the maximum amplitude is attenuated by −13 dB (20% amplitude compared to a broadband amplitude).
- Measured magnetic field amplitudes in the frequency domain at the stimulation frequency and at its harmonics up to 1660 Hz are approximately equal in height. The spectral values decrease due to the system bandwidth of 1660 Hz.
- An increase in the stimulation pulse width from 60 to 120 μs (amplitude and frequency remain the same) results in an increase in spectral values at lower frequencies (even doubling up to 1 kHz), while the main magnitude loop in the spectrum comes closer to the origin and higher. The maximum amplitude in the time domain also increases with increasing stimulation pulse width. The same behavior applies to the maximum amplitude in the time domain.

The same behavior between the measured and the normalized fields at 102 points around the phantom could be observed whether the maximum amplitude in the time domain or a single spectral amplitude at one of the DBS frequencies (stimulation frequency or its harmonics) was considered. Therefore, from the methodological perspective of electrode localization, it makes no difference with which bandwidth and which frequency component is measured, but care should be taken to measure at a lower frequency (e.g., components between 130 Hz and 3 kHz for 60 μs pulse width or between 130 Hz and 1.5 kHz for 120 μs pulse width), because the height of the magnetic field slowly decreases with respect to frequency and its dependence on stimulation pulse width. Of course, this only applies to a sensor that has sufficient sensitivity. Otherwise, however, the signal amplitudes become drastically smaller with decreasing bandwidth and thus the detection limit of the sensor must be significantly improved. The minimum required detection limit depends on the strength of the magnetic field to be detected, which in turn depends on various factors such as measurement distance to the electrode, bandwidth of the magnetic sensor, stimulation amplitude, and stimulation pulse width. The stimulation parameters are already given in a patient (1.5 to 3 mA and 60 μs), even if the amplitude and pulse width can be slightly increased for a short measurement period. Since all magnetic fields around the patient’s head should be measured for precise electrode localization and especially for the determination of electrode rotation, 15 cm was taken as a reference value for the distance. Thus, the required detection limit depends mainly on the bandwidth of the magnetic sensor. For example, a LOD of at least 0.04 pT/Hz${}^{0.5}$ is required for 3 mA, 130 Hz, and 60 μs stimulation if the sensor has an operating bandwidth that captures only the fundamental frequency component of the stimulation signal. The LOD requirement drops by an order of magnitude (to 0.4 pT/Hz${}^{0.5}$) for a sensor with a 1 kHz bandwidth and by approximately two orders of magnitude (to 3 pT/Hz${}^{0.5}$) for a 10 kHz bandwidth. In addition, a longer measurement duration can reduce the noise of the sensor by averaging (SNR increases with the square root of the number of averages when the noise is white noise), so that for a 10-minute measurement (easily tolerable for a patient), a LOD of 10 pT/Hz${}^{0.5}$ is sufficient for a narrow-band sensor which is sensitive only at the fundamental frequency (100 pT/Hz${}^{0.5}$ for a 1 kHz bandwidth, and 1 nT/Hz${}^{0.5}$ for a 10 kHz bandwidth). Further digital signal processing can improve the SNR. Here we can mention the matched filter, which is a method to detect a known signal that is embedded in noise [31,32]. The stimulation signal is well-known since it is set in advance on the neurostimulator. The filter maximizes the SNR of the stimulation signal being detected with respect to the noise. However, this was not part of the work presented at this time.

In addition to the sensor requirements that have been investigated in this correspondence, there are often common demands on sensors, such as operation at ambient temperature without cooling, reduced sensor dimensions, low-power consumption, and high dynamic magnetic field range. However, these are not essential for the measurement of the DBS magnetic field. Therefore, the standard conditions that have been used for the sensors in our experiment suggest that the development of a non-radiative and non-invasive method that is capable of precise determination of the position and rotation of a DBS electrode are suitable, and represent an important development in DBS therapy.

## 5. Conclusions

This work has investigated the minimum limit of detection (LOD) a magnetic sensor must have in order to be used to measure the magnetic field generated by DBS. In order to determine this, magnetic measurements were performed with a state-of-the-art SQUID-based MEG scanner and analyzed. The results have shown that the required LOD depends mainly on the frequency bandwidth of the sensor and on the measurement duration, since other factors, such as the applied DBS stimulation parameters and the measurement distances to the electrode, are predetermined. For a narrow-band sensor that is sensitive only at the stimulation frequency or at its corresponding harmonics, a LOD of at least 0.04 pT/Hz${}^{0.5}$ is required for 3 mA and 60 μs stimulation. This LOD value increases by an order of magnitude for a sensor with a 1 kHz bandwidth and by approximately two orders of magnitude for a broadband sensor with a 10 kHz bandwidth. With recording times of a few minutes and averaging, this value can be increased by another two orders of magnitude.

## Figures and Tables

**Table 1:** Tabulation of the MEG measurements.

Num.	Configuration	Contacts: (−) vs. (+)	Amplitude [mA]	Pulse [μs]	Frequency [Hz]
1	Bipolar	1 vs. 234	3	60	130
2	Bipolar	234 vs. 567	3	60	130
3	Bipolar	567 vs. 8	3	60	130
4	Bipolar	1 vs. 234	1.5	60	130
5	Bipolar	234 vs. 567	1.5	60	130
6	Bipolar	567 vs. 8	1.5	60	130
7	Monopolar	1 vs. Case	1.5	60	130
8	Monopolar	234 vs. Case	1.5	60	130
9	Monopolar	8 vs. Case	1.5	60	130

**Table 2:** The measured magnetic flux densities from all MEG sensors.

Config.	Ampl. [mA], Pulse [μs], Freq. [Hz]	Available Magnetic Flux Densities [pT] within Bandwidth							
		0–10 kHz		0–1660 Hz		0–1 kHz		130 Hz	
		Min	Max	Min	Max	Min	Max	Min	Max
Bipolar	3.0, 60, 130	3.8	30	0.8	6.6	0.5	3.9	0.036	0.38
	1.5, 60, 130	1.9	15	0.4	3.3	1.9	10	0.018	0.19
Monopolar	1.5, 60, 130	-	-	5	195	-	-	1	20

## Data Availability

Not applicable.
